# Stochastic specification of primordial germ cells from mesoderm precursors in axolotl embryos

**DOI:** 10.1242/dev.105346

**Published:** 2014-06

**Authors:** Jodie Chatfield, Marie-Anne O'Reilly, Rosemary F. Bachvarova, Zoltan Ferjentsik, Catherine Redwood, Maggie Walmsley, Roger Patient, Mathew Loose, Andrew D. Johnson

**Affiliations:** 1 School of Life Sciences, University of Nottingham, Queens Medical Centre, Nottingham NG7 2UH, UK; 2 Department of Cell and Developmental Biology, Weill Medical College of Cornell University, New York, NY 10065, USA; 3 Molecular Haematology Unit, Weatherall Institute of Molecular Medicine, Oxford University, Oxford OX3 9DS, UK

**Keywords:** Evolution, Primordial germ cell, PGC, Axolotl, Germ plasm, Mesoderm, Pluripotency

## Abstract

A common feature of development in most vertebrate models is the early segregation of the germ line from the soma. For example, in *Xenopus* and zebrafish embryos primordial germ cells (PGCs) are specified by germ plasm that is inherited from the egg; in mice, *Blimp1* expression in the epiblast mediates the commitment of cells to the germ line. How these disparate mechanisms of PGC specification evolved is unknown. Here, in order to identify the ancestral mechanism of PGC specification in vertebrates, we studied PGC specification in embryos from the axolotl (Mexican salamander), a model for the tetrapod ancestor. In the axolotl, PGCs develop within mesoderm, and classic studies have reported their induction from primitive ectoderm (animal cap). We used an axolotl animal cap system to demonstrate that signalling through FGF and BMP4 induces PGCs. The role of FGF was then confirmed *in vivo*. We also showed PGC induction by Brachyury, in the presence of BMP4. These conditions induced pluripotent mesodermal precursors that give rise to a variety of somatic cell types, in addition to PGCs. Irreversible restriction of the germ line did not occur until the mid-tailbud stage, days after the somatic germ layers are established. Before this, germline potential was maintained by MAP kinase signalling. We propose that this stochastic mechanism of PGC specification, from mesodermal precursors, is conserved in vertebrates.

## INTRODUCTION

The germ cell lineage is established during development through the specification of primordial germ cells (PGCs). Work with diverse animal models indicates that PGC specification is among the earliest cell fate decisions in embryogenesis. Nonetheless, at least two very different modes of PGC specification have evolved in the animal kingdom (Nieuwkoop and Sutasurya, 1979, 1981). The first of these is referred to as preformation. Preformation is found in many commonly used animal models. It describes the cell-autonomous specification of PGCs by determinants that are inherited from the egg, known as germ plasm. Evidence from diverse systems shows that germ plasm represses transcription in developing PGCs, inhibiting their ability to respond to somatic inducing cues (Nakamura and Seydoux, 2008; Shirae-Kurabayashi et al., 2011; Venkatarama et al., 2010). As a result, presumptive germ cells are segregated from somatic cells, or soma, in the earliest stages of development. However, preformation is a derived trait that evolved by convergence, and the acquisition of germ plasm is associated with embryological innovations that enhance evolvability (Crother et al., 2007; Evans et al., 2014; Johnson et al., 2003a,b, 2011). The conserved mode of PGC specification, however, is called epigenesis, which describes a process wherein PGCs are induced from pluripotent cells by extracellular signals (Johnson et al., 2003a,b, 2011). To date, however, a detailed mechanism for epigenesis has only been elaborated in mouse.

At around day 6.25 post-coitum of mouse development, the transcription factor Blimp1 (Prdm1 – Mouse Genome Informatics) is induced in about six to eight cells of the proximal epiblast that are precursors to the PGCs (Ohinata et al., 2005). *Blimp1* is part of a tripartite transcription factor network that induces the PGC programme (Magnusdottir et al., 2013; Nakaki et al., 2013). It is also a determinant of the germ line, which acts by repressing somatic gene expression in specified PGC precursors. In the absence of *Blimp1*, nascent PGCs are diverted to a mesodermal cell fate (Kurimoto et al., 2008; Ohinata et al., 2005). Related to this, recent work has shown that *Blimp1* is a direct target of brachyury (*T*), a master regulator of mesoderm development (Aramaki et al., 2013). However, *T* only induces *Blimp1* in the presence of bone morphogenetic protein 4 (Bmp4) signalling. How BMP4 mediates the induction of *Blimp1* to specify PGCs is unknown. But these signals are required early in the process, before the mesoderm programme can be established. Furthermore, it is unknown whether the mechanism of PGC specification that has been identified in mouse is conserved in other vertebrates.

Among vertebrates, epigenesis was first described in classic studies that used urodele amphibians (salamanders). Urodele embryos do not contain germ plasm (Johnson et al., 2001; Tamori et al., 2004), and numerous authors report the induction of PGCs from primitive ectoderm (the animal cap) of axolotls, and other species, in response to signals that induce the ventral mesoderm (Boterenbrood and Nieuwkoop, 1973; Kocher-Becker and Tiedemann, 1971; Maufroid and Capuron, 1977; Michael, 1984; Sutasurja and Nieuwkoop, 1974). This is consistent with the mesodermal origin of urodele PGCs (Bachvarova et al., 2004; Humphrey, 1925; Johnson et al., 2001; Nieuwkoop, 1947; Smith, 1964), but what these signals are, and how they discriminate PGCs from neighbouring mesodermal cells, is unknown. In this regard, it is uncertain whether PGC precursors are specified by germline determinants (Michael, 1984; Smith et al., 1983), or whether they arise in response to mesoderm patterning.

Here, we show ectopic and *in vivo* induction of PGCs in axolotl embryos by fibroblast growth factor (FGF) signalling, and we confirm the mesodermal origin of PGCs by showing they can also be induced by Brachyury and BMP4. We find no evidence for germline determinants. Rather, mesoderm precursors in the ventral marginal zone (VMZ) are patterned towards the development of PGCs or blood cells by the competing effects of FGF and Nodal signalling, respectively. After this, germline potential is maintained within pluripotent mesodermal cells through MAP kinase (MAPK) activity. Indeed, specified PGC precursors are not irreversibly committed to the germ line until the tailbud stages, days after the somatic germ layers have been established. Based on the phylogenetic positioning of urodeles, we propose that this stochastic mechanism of PGC specification is ancestral to vertebrates.

## RESULTS

### PGCs are derived from pluripotent cells

**Fig. 1.**
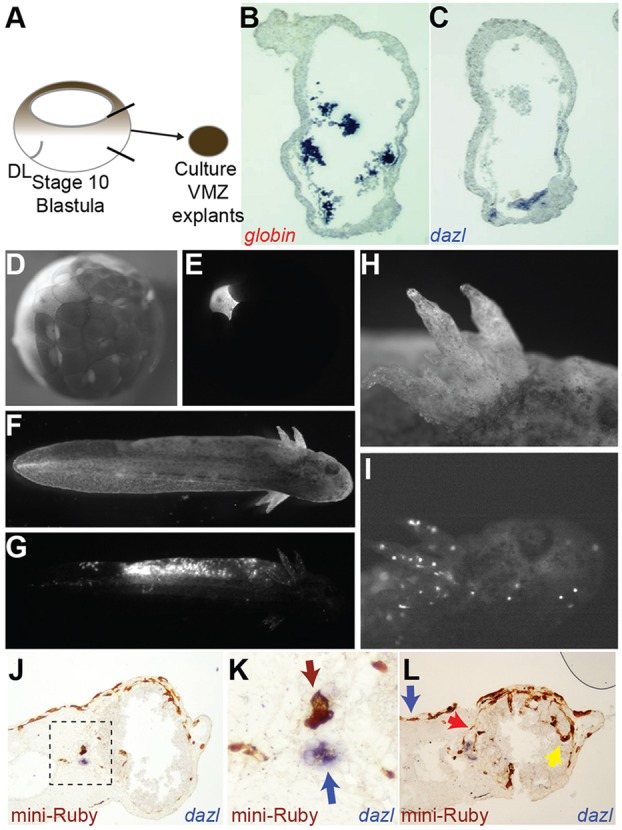
**PGCs develop from pluripotent cells in the VMZ.** (A) Schematic showing the isolation of VMZ explants. DL, dorsal lip of the blastopore. Black lines on ventral side show approximate area of dissection. (B,C) ISH (purple) in sections of the same explant shows expression of *globin* (B) and *dazl* (C). (D) A single blastomere of a 128-cell embryo that had been injected with mini-Ruby shown under bright light. Ventral is to the left. (E) The same embryo as that in D shown under UV light to show labelling of a single blastomere. (F) Bright field view of an embryo at stage 42. (G) Under UV light, the embryo shows labelling by mini-Ruby in lateral mesoderm. (H) View of gills from an injected embryo under bright light. (I) UV light shows blood cells circulating through the gills. (J) Mid-trunk section of an embryo, stained to detect horseradish peroxidase activity of mini-Ruby (brown) and ISH for *dazl* RNA (purple). (K) Close-up of the boxed section in J shows a labelled PGC cluster stained for mini-Ruby HRP activity (brown) from the injected side of embryo (top, brown arrow). ISH for *dazl* (purple) detects a PGC cluster from the uninjected side of the embryo (bottom, blue arrow). (L) A section from further towards the anterior of the same embryo as that shown in K shows mini-Ruby labelling in ectoderm (blue arrow), mesoderm (red arrow) and endoderm (yellow arrow).

The progenitors of PGCs have been previously mapped to the VMZ of gastrulating embryos by using deletion and transplant studies (Nieuwkoop, 1947; Smith, 1964). To verify this, we prepared VMZ explants (Fig. 1A) from mid-gastrula embryos (stage 10.5) and cultured them until stage 42, when expression of the PGC-specific axolotl *dazl* gene can be detected by using *in situ* hybridisation (ISH) (Bachvarova et al., 2004; Johnson et al., 2001). The sectioned explants were hybridised to a probe for *dazl* or axolotl α*-globin* (a marker of blood cells), and we detected expression of both genes in the same explants (Fig. 1B,C). Ventral blood islands (VBI) have a dual origin in *Xenopus* – from both the dorsal and ventral side of the embryo (Ciau-Uitz et al., 2000). To determine the origins of axolotl VBI, we injected RNA coding for β-galactosidase (β-gal) into dorsal or ventral blastomeres at the four-cell stage (supplementary material Fig. S1A,B). These were later (stage 30) stained for β-gal activity and analysed for *globin* expression by whole-mount ISH. The β-gal signal overlapped with *globin* expression in cells from the VMZ, but not those from the dorsal marginal zone. Thus, blood is exclusively of ventral origin in axolotls, and *globin* RNA is an unambiguous marker for somatic derivatives of the VMZ.

We next performed lineage labelling to clarify the origins of germ cells. Individual blastomeres in the ventro-lateral region of embryos at the 128-cell stage were injected with mini-Ruby (Fig. 1D,E). Embryos in which the lateral mesoderm was strongly labelled were selected for analysis (Fig. 1F,G). These embryos also contained circulating blood cells (Fig. 1H,I). The embryos were sectioned and subjected to ISH with a probe against *dazl*, and then stained for horseradish peroxidase activity to detect mini-Ruby. Sections from representative embryos showed that mini-Ruby was present in clusters of PGCs (Fig. 1J,K; supplementary material Fig. S1C). Mini-Ruby was also detected in each of the somatic germ layers (Fig. 1L). These results demonstrate that the cells in axolotl animal caps can produce germ cells and somatic cells, similar to the ground-state potential of the mouse epiblast (Dixon et al., 2010; Nichols and Smith, 2011). Moreover, the large amount of label that was observed in somatic tissue suggests that restriction of the germ line occurs relatively late in axolotl development.

### Induction of PGCs from animal caps by FGF

**Fig. 2.**
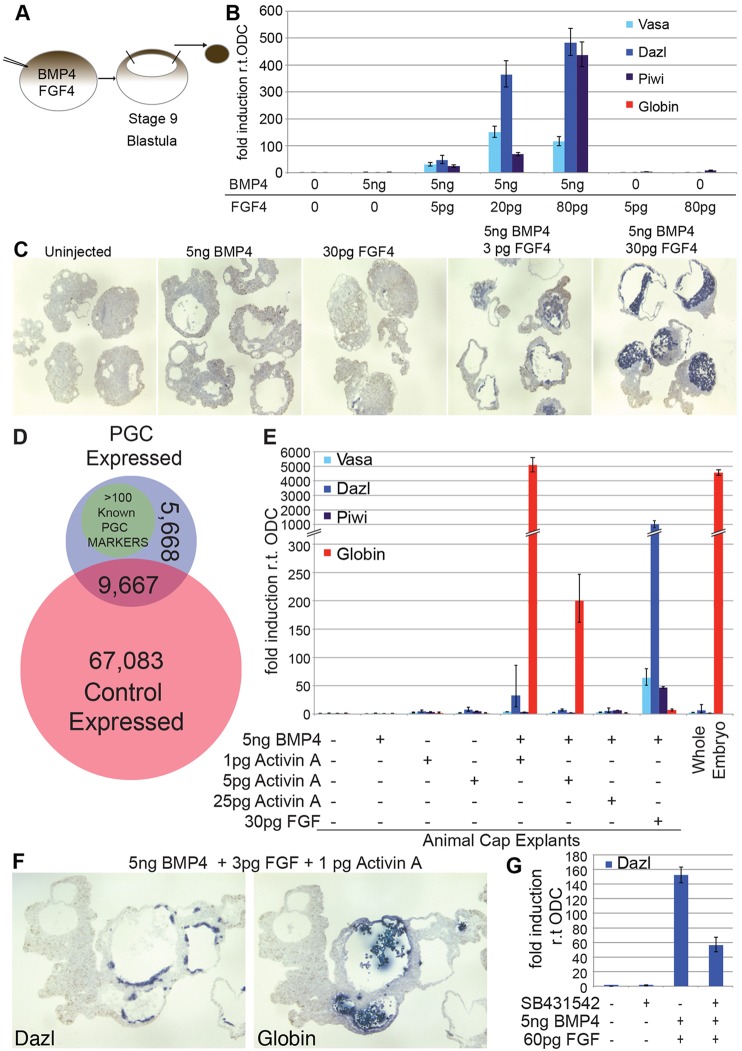
**Ectopic induction of PGCs by FGF and BMP4.** (A) Scheme for PGC in animal cap explants. (B) qRT-PCR analysis of PGC (*dazl*, *vasa*, *piwi*) and blood (*globin*) markers in animal caps that expressed FGF and BMP4. The data were normalised to expression of ornithine decarboxylase. Means±95% CI are shown. (C) ISH to detect *dazl* (purple) in animal caps after injection of the indicated RNAs. (D) Diagram comparing the deep sequencing of animal caps containing PGCs (blue) against that of unstimulated controls (red). (E) qRT-PCR analysis of PGC or blood markers in animal caps that expressed Activin A or FGF with BMP4. The data were normalised to ODC. Means±95% CI are shown. (F) Serial sections of a cap that had been induced with low levels of FGF and Activin A with BMP4 and probed for *globin* or *dazl* expression by using ISH. (G) qRT-PCR analysis of *dazl* expression in caps that had been induced with FGF and BMP4 in the presence or absence of SB431542. The data were normalised to ODC. Means±95% CI are shown.

Based on histological criteria (Nieuwkoop and Sutasurya, 1979), the induction of PGCs has been reported from urodele animal caps that were either cultured with cells from the vegetal hemisphere (Boterenbrood and Nieuwkoop, 1973; Michael, 1984; Sutasurja and Nieuwkoop, 1974) or treated with extracts from chicken embryos (Kocher-Becker and Tiedemann, 1971). Boterenbrood and Nieuwkoop (Boterenbrood and Nieuwkoop, 1973) have shown that maximal induction was achieved upon culture with ventral vegetal cells of early gastrulae. In *Xenopus*, these cells express FGF and BMP4 (Hemmati-Brivanlou and Thomsen, 1995; Slack et al., 1987). Therefore, we tested whether these molecules participate in PGC induction, and we used the expression of PGC-specific markers as an assay system. In contrast to the effects of BMP4 in mouse embryos, the forced expression of BMP4, over a wide range of RNA concentrations, was insufficient to induce PGC-specific gene expression in animal caps. Rather, similar to uninjected caps (data not shown), these cells differentiated as epidermis. We tested varying amounts of RNA encoding *Xenopus* embryonic FGF (eFGF; FGF4 – Xenbase; termed FGF herein), with or without RNA encoding *Xenopus* BMP4. The embryos were injected at the one-cell stage, and then the animal caps were explanted at midblastula stage (stage 9) (Fig. 2A) and cultured for about 12 days, until stage 42 (early larva). The RNA was then analysed by using reverse transcription and quantitative PCR (qRT-PCR). In response to FGF and BMP4, the animal caps showed strong induction of *dazl*, *vasa* and *piwi* (Fig. 2B). These markers of PGCs showed a dose-dependent response to FGF, and the highest expression was observed at maximal input. The maximum level of induction also required the highest dose of BMP4 that we tested (5 ng) (data not shown), and the expression of PGC markers was not detected in response to FGF alone. We confirmed these findings by using ISH analysis on sectioned animal caps (Fig. 2C). These results showed that all of the animal caps that had been induced by using FGF and BMP4 contained *dazl*-expressing cells, and more cells expressed *dazl* upon injection of higher levels of FGF, confirming the dose-dependent response.

The cell morphology of animal caps that had been injected with RNA encoding FGF and BMP4 (programmed) were then examined by staining sections with Toluidine Blue (supplementary material Fig. S2A,B). The staining of cells from these animal caps was then compared with the staining of native PGCs in the genital ridge of intact embryos (supplementary material Fig. S2C-F). PGCs contain a high nuclear to cytoplasmic volume, pigment granules, abundant mitochondria and a single large nucleolus (Nieuwkoop and Sutasurya, 1979). These features were readily detected in the PGCs of embryos and the cells of the programmed animal caps. By using deep sequencing, we analysed the RNA expression profiles of the animal caps that had been prepared under the different conditions. This analysis identified >100 known PGC markers that were expressed only in caps that had been induced with FGF and BMP4 (Fig. 2D; supplementary material Table S2). We could not demonstrate that the PGCs that had been induced were capable of germline transmission; nevertheless, the data strongly suggest that FGF and BMP4 are sufficient to stimulate normal PGC development. Moreover, our chosen markers are reliable indicators of PGC induction. However, because precursors of PGCs and blood develop in close proximity in the VMZ (Nieuwkoop, 1947; Fig. 1), we were surprised that *globin* RNA was undetectable in the animal caps that contained PGCs; therefore, we aimed to identify conditions that would induce the development of blood cells.

Nodal signalling initiates mesoderm development in vertebrates and is mimicked experimentally by Activin A. We injected embryos with varying amounts of RNA that encoded *Xenopus* Activin A and a constant amount of RNA that encoded BMP4 (5 ng), we then analysed gene expression in the animal caps. Low Activin A input (1 pg RNA) resulted in massive *globin* induction (Fig. 2E). This was diminished at fivefold-higher levels of Activin A and it was eliminated at high concentrations (25 pg), which induce endoderm (data not shown). Thus, *globin* induction reflects tissue patterning. However, PGC markers were undetectable in animal caps that had been induced by expression of Activin A. We then used low levels of Activin A (1 pg) or FGF (3 pg), concomitant with BMP4 (5 ng). We examined these caps by using ISH and found that at low levels of FGF *globin* and *dazl* can be co-induced, albeit in separate domains (Fig. 2F). Taken together, these data demonstrate the mutually exclusive effects of FGF and Activin A, which induce PGCs or blood cells, respectively. To test whether PGC specification requires Nodal signals that are downstream of FGF, we treated animal caps that expressed FGF and BMP4 with the Nodal signalling inhibitor SB431542 (Swiers et al., 2010). Under these conditions, the expression of PGC markers was induced at about 50% of the level of that without inhibitor (Fig. 2G). Thus, FGF is sufficient to stimulate PGC production in the presence of BMP4.

### *In vivo* induction of PGCs by FGF

**Fig. 3.**
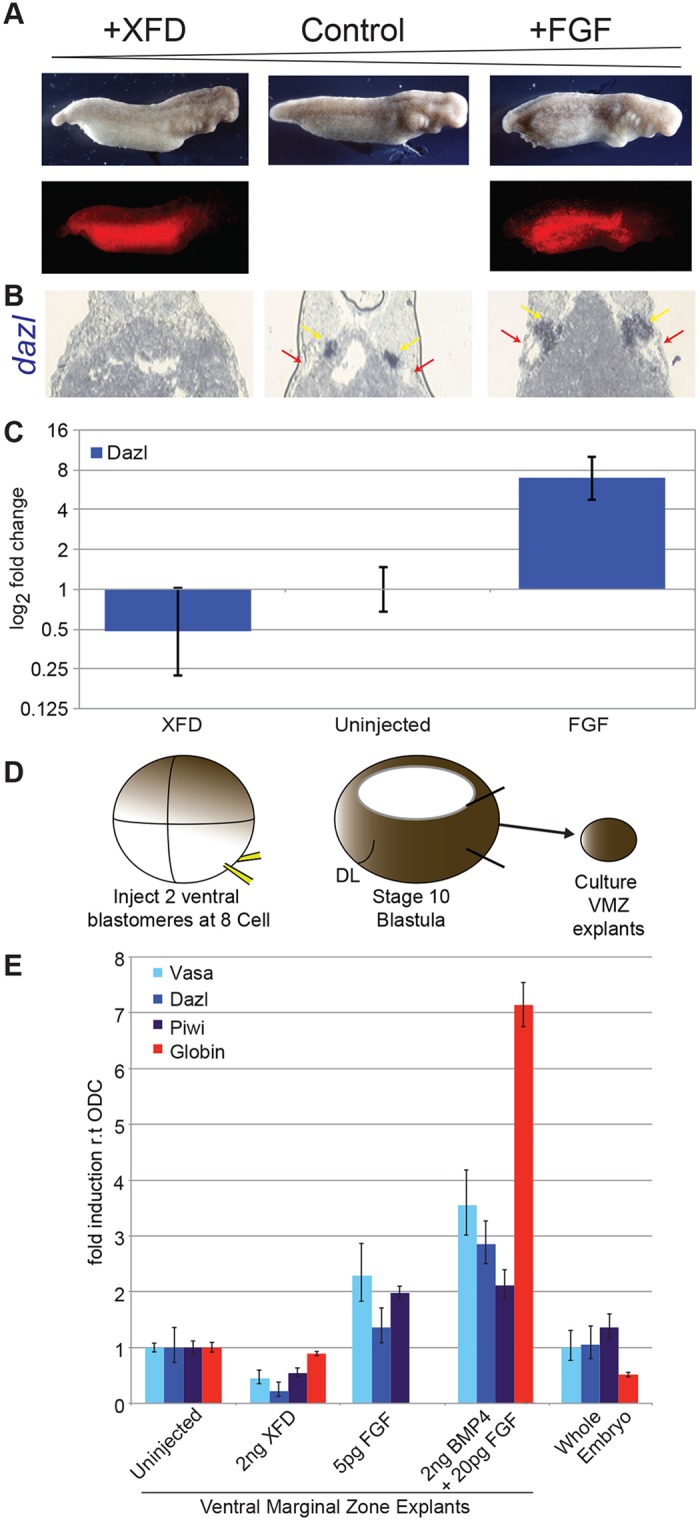
**FGF regulates PGC induction *in vivo*.** (A) The top row of images shows the effects of FGF signalling on posterior development of the axolotl. The control was uninjected. The bottom row of images shows embryos under UV light to detect mini-Ruby. XFD, dominant-negative FGF receptor. (B) ISH to detect *dazl* in sections from embryos as described in A. Alignment of the images also corresponds to the labelling in A. Mesonephric ducts are indicted by red arrows, PGCs by yellow arrows. (C) qRT-PCR analysis of *dazl* expression in whole embryos with altered FGF signalling. The fold-change relative to uninjected controls is shown. Means±95% CI are shown. (D) Scheme for isolation of VMZ explants. DL, dorsal lip of the blastopore. (E) qRT-PCR analysis of PGC and blood marker expression in embryos, or explants from embryos that had been treated as indicated. Data were normalised to ODC. Means±95% CI are shown.

We next asked whether FGF signalling is required for the induction of PGCs *in vivo*. For this, we altered signalling within the VMZ. We injected both ventral vegetal blastomeres of eight-cell-stage embryos with RNA that encoded either a dominant-negative FGF receptor (XFD; 5 ng) (Amaya et al., 1991) or FGF (20 pg). We included mini-Ruby as a lineage tracer. Fig. 3A shows representative embryos from such an experiment at stage 35. Expression of XFD caused posterior truncations, reminiscent of its effect in *Xenopus* (Amaya et al., 1991). Conversely, overexpression of FGF expanded the posterior domain. Sections from injected embryos and controls, at stage 42, were analysed by using ISH for *dazl* expression (Fig. 3B). Expression of XFD eliminated *dazl* RNA expression. Importantly, axolotl PGCs develop in close proximity to the mesonephric ducts (MD) (Bachvarova et al., 2004; Nieuwkoop, 1947). Consistent with this, expression of XFD also eliminated development of the MD. By contrast, overexpression of FGF expanded the PGC domain, relative to controls, and this treatment expanded the MD region. These results suggest that FGF signalling mediates the development of the intermediate mesoderm, within which PGC precursors develop; therefore, we investigated the effects on PGCs by performing qRT-PCR on whole embryos. This showed that the level of *dazl* RNA was increased about sixfold by elevated levels of FGF (Fig. 3C), whereas XFD reduced *dazl* expression to background levels, i.e. below that of uninjected controls.

We then analysed the effects of altered FGF signalling in VMZ explants. This regime controls for effects that are caused by disrupted morphogenetic movements. Ventral vegetal blastomeres were, again, injected with RNA, but then the VMZs were dissected at stage 10.5 and cultured until stage 42 (Fig. 3D). Similar levels of *globin* RNA, and of PGC markers, were detected in control explants and intact embryos (Fig. 3E), demonstrating that dissections included the entire VMZ. Expression of XFD, again, diminished the expression of PGC markers, without affecting those of blood. Also, elevated levels of FGF enhanced the expression of PGCs, and downregulated the expression of *globin*, the latter resembling the effects of FGF in *Xenopus* (Isaacs et al., 2007; Walmsley et al., 2008). However, RNAs encoding both BMP4 and FGF increased the expression of markers of both cell types. Because BMP signalling is required for the induction of PGCs and blood, this result suggests that the levels of endogenous BMP limit the production of these cell types in the VMZ. From this, and the work above, we postulated that FGF and Nodal signalling pathways compete for a common pool of BMP-dependent precursors; therefore, we tested this hypothesis in animal caps.

### Stochastic specification of PGCs

**Fig. 4.**
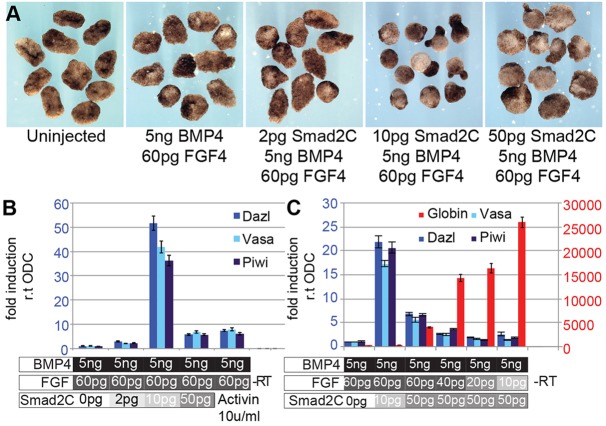
**Ectopic induction of PGCs by FGF and SMAD2.** (A) Morphology of animal caps expressing FGF and constitutively active Smad2 (Smad2C) with BMP4. (B) qRT-PCR analysis showed that a low level of Smad2C RNA enhances PGC induction by FGF. Data were normalised to the expression in caps that had been injected with RNA encoding FGF and BMP4. Means±95% CI are shown. (C) qRT-PCR analysis to detect PGC or blood markers in response to titration of FGF and Smad2C in the presence of a constant amount of RNA encoding BMP4. The numbers in red on the right-hand side of the graph show the fold-change in *globin* expression. Means±95% CI are shown. –RT, no reverse transcriptase.

We titrated the FGF and TGFβ signalling pathways against one another, under conditions in which BMP signals were not limiting. Having shown that 5 ng of RNA encoding BMP4 was sufficient for maximal induction of PGCs or blood cells from animal caps, this level of BMP signalling was kept constant. To initiate TGFβ signalling, we employed a constitutively active variant of Smad2 (Smad2C), which translocates to the nucleus independently of receptor stimulation (Howell and Hill, 1997). We expressed increasing amounts of Smad2C in animal caps with constant levels of FGF and BMP4 (Fig. 4A). However, we did not observe the expected inhibition of the development of PGCs; instead, the introduction of RNA encoding Smad2C (10 pg) increased the expression of PGC markers (Fig. 4B). We then titrated FGF against Smad2C and a clear pattern emerged (Fig. 4C): as the levels of RNA encoding Smad2C were increased to 50 pg, *globin* expression became detectable, but with a corresponding decrease in the expression of PGC markers. Then, by reducing FGF levels (10-40 pg RNA) and keeping Smad2C constant at 50 pg, *globin* expression was increased, and PGC markers were decreased. These data strongly suggest that the pathways that are stimulated by FGF and TGFβ compete for BMP-dependent precursors, and from this PGCs or blood cells are differentially specified. Moreover, the increased PGC production that is stimulated by increased Smad2C expression suggests that the mesodermal precursors that are specified by Smad2 can be directed by FGF towards PGC development. These results demonstrate the stochastic nature of PGC specification in axolotls. In addition, they led us to consider a role for Brachyury in PGC specification, because it specifies mesoderm precursors and is cooperatively induced by Activin A and FGF (Cunliffe and Smith, 1992; Latinkic et al., 1997; supplementary material Fig. S3A).

### Brachyury induces PGCs

**Fig. 5.**
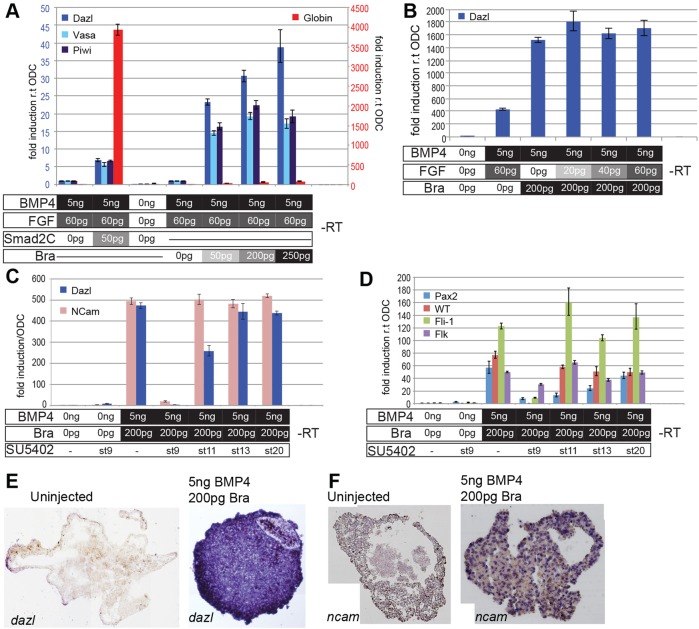
**Induction of PGCs by *Brachyury*.** (A) qRT-PCR analysis of gene expression in caps that had been programmed with a constant amount of FGF and BMP4, with Smad2C or increasing amounts of Brachyury (Bra). Scales are at different levels; blue scale is PGC markers, red is *globin.* (B) qRT-PCR analysis of *dazl* expression in caps that had been programmed with constant levels of Brachyury and BMP4 with increasing levels of FGF. (C) qRT-PCR of *dazl* and *ncam* expression in caps expressing Brachyury and BMP4 after timed addition of SU5402 [stage (st) 9, 11, 13 or 20]. (D) qRT-PCR of AGM markers from the same caps as those used in C. All qRT-PCR data were normalised to ODC. –RT, no reverse transcriptase. Means±95% CI are shown. (E) ISH to detect *dazl* RNA (purple) in caps that had been induced with Brachyury and BMP4, or in caps from uninjected embryos. Note the vesicle from *dazl*-negative cells (arrow). (F) ISH to detect *ncam* RNA (purple) in caps prepared as described in E.

We co-injected increasing amounts of RNA that encoded axolotl Brachyury, along with constant levels of RNA encoding FGF and BMP4. Increasing the levels of Brachyury led to increased expression of PGC markers (Fig. 5A), but they did not induce *globin*. Then we tested the effects of the expression of Brachyury and BMP4 in the absence of RNA encoding FGF, and *dazl* expression was still induced at high levels (Fig. 5B), again, there was a lack of expression of *globin*. Importantly, in the absence of BMP4, Brachyury did not induce *dazl* (supplementary material Fig. S3B), indicating that Brachyury does not target PGC markers directly; rather, it cooperates with BMPs to induce PGCs.

Recent work has shown that, in mouse embryos, *Blimp1* is a direct target of *T*, but that *Blimp1* is only activated in the presence of BMP signalling during PGC specification (Aramaki et al., 2013). However, using whole-mount ISH, we could first detect *blimp1* expression at about stage 15 (neurula), but the expression was found only in the developing neural folds; *blimp1* was not expressed in lateral mesoderm (supplementary material Fig. S4A). We then found that neither FGF nor Brachyury induced *blimp1* in animal caps, regardless of the inclusion of BMP4 (supplementary material Fig. S4B,C). However, Brachyury and BMP4 did induce the expression of neural cell adhesion molecule (*ncam*) (Fig. 5C), as well as markers that are expressed in the MD (*wt1*, *pax2*; Carroll and Vize, 1996; Fleming et al., 2013) and the dorsal aorta (*fli1*, *flik1*; Ciau-Uitz et al., 2000) (Fig. 5D). These structures, together with the genital ridge, comprise the aorta-gonad-mesonephros (AGM) region (Medvinsky and Dzierzak, 1996). Herein, these genes are referred to as somatic AGM markers. Taken together, the data suggest that Brachyury and BMP4 do not induce lineage-restricted PGC precursors, in contrast with their roles in mouse. Instead, PGCs might arise from pluripotent mesoderm precursors in response to patterning signals. To test this, we asked whether the expression of PGCs and AGM markers was dependent on FGF signalling, which is activated downstream of *brachyury* in *Xenopus* (Isaacs et al., 1994; Schulte-Merker and Smith, 1995). Indeed, we found that when animal caps that had been programmed with Brachyury and BMP4 were incubated with the FGF signalling inhibitor SU5402 (50 μM) before the onset of gastrulation, the expression of both PGC and somatic AGM markers was abolished (Fig. 5C,D). By contrast, the addition of the inhibitor after gastrulation had no effect. Thus, FGF acts downstream of *brachyury* and is required during gastrula stages in order to specify the PGC lineage.

A requirement for FGF notwithstanding, we reasoned that *brachyury* acts in a cell-autonomous manner in order to specify PGCs, and that PGCs should, therefore, show a different pattern of distribution to that in animal caps that had been induced with FGF and BMP4. To assess this, we analysed caps that had been programmed with Brachyury and BMP4 for *dazl* expression by using ISH. *dazl*-positive cells were distributed evenly throughout all of the programmed caps (ten embryos), but not the caps from uninjected control embryos (zero out of ten embryos) (Fig. 5E). The programmed caps, however, also contained somatic vesicular structures comprised of cells that did not express *dazl* (arrow). We then examined the caps from programmed sibling embryos for the expression of *ncam* as a somatic cell marker. *ncam*-positive cells were found in programmed caps in a punctate pattern, quite different from that of the uniform pattern we observed for *dazl* (Fig. 5F). These results confirm that both PGCs and somatic cells are derived from mesoderm precursors that are specified by Brachyury and BMP4. We then investigated when lineage restricted PGCs are established.

### Germline restriction occurs after gastrulation

**Fig. 6.**
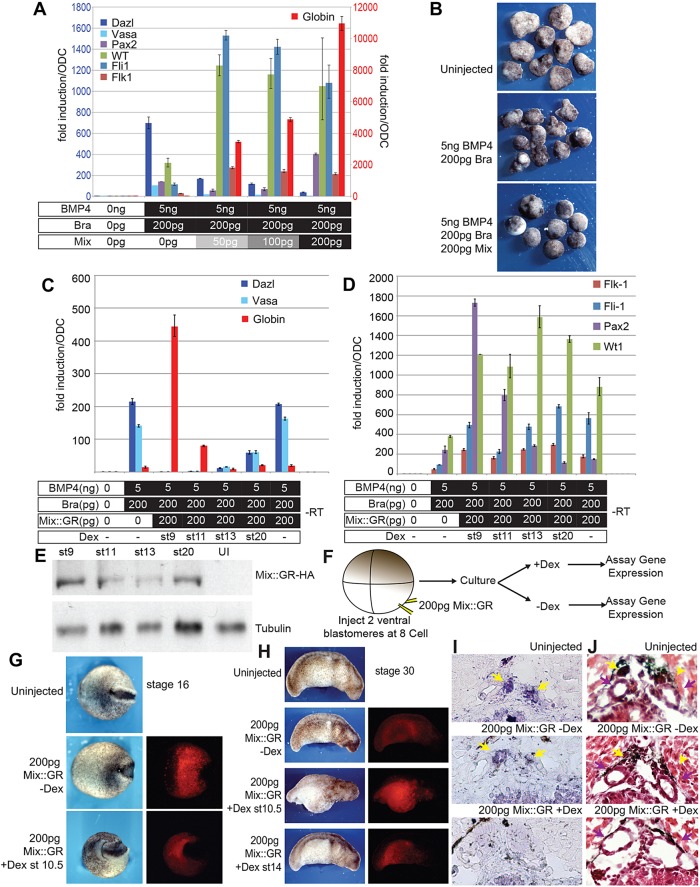
**Mix redirects specified PGC precursors to somatic development.** (A) qRT-PCR analysis of markers for PGCs or somatic cells in caps that had been programmed with Brachyury (Bra) and BMP4 and increasing levels of Mix. The *y*-axis scales are at different levels. The red scale is for *globin*. (B) The morphology of caps in response to Mix. (C) qRT-PCR analysis of PGC and somatic markers after timed activation of Mix-GR-HA (Mix::GR) in caps that had been programmed by Brachyury and BMP4. Dex, dexamethasone. (D) qRT-PCR analysis of the response of AGM markers to Mix. St, stage. For all qRT-PCR data, Means±95% CI are shown. (E) Western blot analysis to detect the Mix-GR-HA fusion protein in staged caps by using an antibody against HA. (F) Experimental design of ventral Mix induction as shown in G-J. Embryos were injected at the VMZ with RNA encoding Mix-GR-HA and mini-Ruby; Dex was administered at specific time points. (G) Embryos that had been injected with the indicated RNAs were examined at the neurula stage under bright-field (left) or UV (right) conditions. (H) Embryos from G at the tailbud stage under a bright field (left) or UV (right). (I) ISH for *dazl* RNA (purple) in stage-45 control embryos, or after Mix-GR-HA had been activated at stage 14. Yellow arrows indicate PGCs. (J) H&E-stained sections of embryos that had been treated as described in H. PGCs are indicated by yellow arrows. Purple arrows indicate mesonephric ducts.

*Mix* is a downstream effector of *nodal* signalling (Swiers et al., 2010), and we reasoned that it might be capable of competing with the effects of *brachyury.* In this context, we hoped to use *mix* to inhibit PGC development. We expressed increasing levels of axolotl Mix (herein referred to as Mix) in animal caps, along with a fixed amount of Brachyury and BMP4. As expected, PGC marker expression was diminished in response to increasing levels of Mix (Fig. 6A). Conversely, Mix induced somatic markers of the AGM, as well as *globin*, and the levels of *globin* showed a dose-dependent response to the levels of Mix. At the highest concentrations of Mix (200 pg), *globin* expression reached a maximum, and PGC markers were undetectable. Mix also changed the morphology of the caps so that they appeared smoother and rounder, resembling the cap morphology that was induced by Activin A (Fig. 6B). These data demonstrate that *mix* is sufficient to interfere with PGC development, and we reasoned that its conditional activation might provide a system to investigate the timing of germline restriction.

We fused the coding region of Mix to a glucocorticoid receptor (GR) and a haemagglutinin (HA) tag (Mix-GR-HA). Protein from this construct is retained in the cytoplasm until the addition of the glucocorticoid dexamethasone (Tada et al., 1997), and the HA epitope allows detection of the fusion protein. Mix-GR-HA was co-expressed in animal caps with Brachyury and BMP4. The fusion protein was then activated at various times by addition of dexamethasone, and the animal caps were assayed for gene expression. Application of dexamethasone at the midblastula stage eliminated PGC marker expression, and *globin* expression was induced strongly (Fig. 6C), similar to the effects that are observed upon the expression of native Mix. The application of dexamethasone at this stage also induced *ncam* and AGM markers (Fig. 6D). When activated after the gastrula stage, Mix also eliminated PGC markers, although *globin* induction was not as strong. Indeed, *globin* was not induced if Mix was activated after gastrulation, but this did not compromise the expression of AGM markers. Western blot analysis showed that the fusion protein was stable throughout the course of these experiments (Fig. 6E); therefore, changes in the ability of the fusion protein to induce expression of the different markers were not attributable to diminished levels of the protein. Most importantly, PGC markers first show resistance to Mix expression at (approximately) the tailbud stage (stage 20), suggesting that germline commitment is initiated at a point between the neurulation and tailbud stages. Because somatic cells are specified during gastrulation, these data suggest that germline restriction occurs after the somatic lineages are established. This is the opposite to the observed sequence of events in other animal models.

We then tested this hypothesis *in vivo.* We co-injected RNA encoding Mix-GR-HA into ventral vegetal blastomeres with mini-Ruby (Fig. 6F), and then isolated embryos that had been properly targeted. These were treated with dexamethasone at different stages. When dexamethasone was added to early gastrulae (stage 10.5), gastrulation movements were disrupted, resulting in hyper-ventralised embryos (Fig. 6G,H). However, when dexamethasone was administered after gastrulation (stage 14), the embryos developed normally. These embryos were then cultured until stage 45, when they were examined for *dazl* expression by using ISH (Fig. 6I). d*azl* expression in most embryos (three out of five) was undetectable, indicating loss of PGCs. Importantly, when sections from sibling embryos were stained with Haematoxylin and Eosin (H&E), it was clear that the entire genital ridge was eliminated, with an associated expansion of the MD (Fig. 6J). This phenotype was not observed in the absence of dexamethasone.

Our results suggest that PGCs are derived from uncommitted cells in the intermediate mesoderm. Given that the transcription factor *osr1* is expressed in undifferentiated intermediate mesoderm (Rankin et al., 2012; Wang et al., 2005), we investigated whether the expression of *osr1* correlated with the lineage restriction of PGCs. In a timecourse analysis of animal caps that had been programmed with FGF and BMP4, we found that *osr1* was expressed until the mid-tailbud stage (stage 25), when it began to be downregulated. This is approximately the same stage at which *dazl* expression commences (supplementary material Fig. S5), and it is consistent with our data on the timing of germline restriction.

### MAPK signalling is required for PGC development

**Fig. 7.**
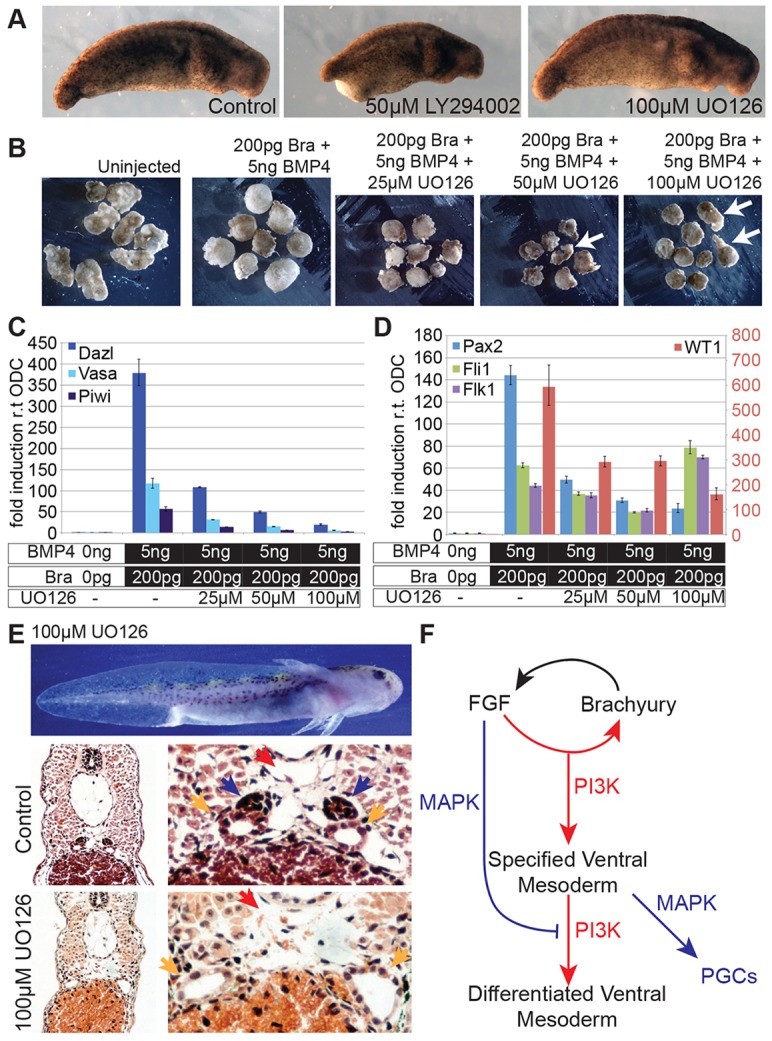
**MAPK signalling is required for PGC development.** (A) Embryos at stage 30 after treatment with UO126 (100 μM) or LY294002 (50 μM) during gastrula stages. (B) Morphology of caps that expressed Brachyury (Bra) and BMP4 after treatment with varying doses of UO126. Elongation is indicated by arrows. (C) qRT-PCR of PGC markers from animal caps that expressed Brachyury and BMP4 and had been treated with increasing levels of UO126. (D) Expression of AGM markers from the caps shown in C. Data were normalised to ODC. The red numbers are the fold induction of WT1. Means±95% CI are shown. (E) Top panel: stage-45 embryo after treatment with UO126 (100 μM) during gastrula stages. Middle panel: H&E-stained section of AGM from an untreated stage-45 embryo. PGCs have darkly stained nuclei and are indicated by blue arrows. Mesonephric ducts are indicated by yellow arrows and the dorsal aorta by the red arrow. Bottom panel: section of AGM from a stage-45 embryo that had been treated with UO126 (100 μM) during gastrula stages. In the middle and bottom rows, the image on the right is an enlarged section of that shown on the left. (F) Model for mesoderm patterning in the VMZ. FGF is activated downstream of Brachyury. PI3K promotes mesoderm development. MAPK signalling inhibits mesoderm differentiation to promote the development of PGCs.

In the data presented above, we showed that abrogation of FGF signalling, by using XFD, caused a posterior truncation, similar to that observed in *Xenopus* (Amaya et al., 1991). This resulted in the elimination of PGCs, as well as the MD. To better understand the role of FGF in PGC development, we investigated the events that occur downstream of receptor stimulation. First, we used the small-molecule signalling inhibitor LY2941002, which inhibits phosphoinositide 3-kinase (PI3K) signalling. LY2941002 (50 μM) was applied before the midblastula transition (MBT) and then washed away after gastrulation was completed (stage 14). Embryos treated in this way showed a posterior mesoderm truncation (20 out of 20) (Fig. 7A). These results were expected, based on the effects of PI3K inhibition in *Xenopus* embryos (Carballada et al., 2001), and they demonstrate that PI3K inhibition is sufficient to phenocopy the effects of the expression of XFD. We then tested the effects of MAPK signalling inhibition using U0126, a soluble inhibitor of MAPK activation. We placed embryos in medium that contained UO126 at levels as high 100 μM, which were sufficient to completely inhibit FGF-mediated activation of MAPK (supplementary material Fig. S6A). Embryos were treated with the inhibitor from the MBT through to the gastrula stages, as described above; however, we did not observe any morphological defects. Indeed, all UO126-treated embryos (*n*=25) remained indistinguishable from untreated controls (Fig. 7A). This result was unexpected because inhibition of MAPK in *Xenopus* embryos causes gastrulation defects (Gotoh et al., 1995; Sivak et al., 2005; Umbhauer et al., 1995). We then considered a role for MAPK signalling in PGC development.

We overexpressed Brachyury and BMP4 in embryos, and then incubated the animal caps in medium that contained increasing concentrations of U0126 through the gastrula stages. MAPK inhibition induced a morphological response, such that, at high concentrations of the inhibitor, the caps showed an elongation response (Fig. 7B). More importantly, the expression of PGC-specific genes was decreased in response to the increasing doses of U0126, and, at the highest concentration of U0126 (100 μM), the expression of these genes was reduced to background levels (Fig. 7C). By contrast, AGM markers were not eliminated (Fig. 7D). Interestingly, *osr1* expression was abolished by the inhibition of MAPK (supplementary material Fig. S6B), suggesting that loss of MAPK signalling causes the premature differentiation of the intermediate mesoderm.

Given these data, we revisited the effect of MAPK inhibition on intact embryos. Embryos were, again, incubated in medium containing UO126 (100 μM) during the cleavage stages through to gastrulation (stage 13) and were then thoroughly washed with normal medium. These embryos were then cultured for an additional two weeks, in the absence of the inhibitor, until stage 45. The external phenotype of the treated embryos at this stage was indistinguishable from that of controls (Fig. 7E). The embryos were then harvested, and sections from the treated and control embryos were stained with H&E to examine internal structures. Startlingly, most of the treated embryos completely lacked a genital ridge and PGCs (five out of six embryos) (Fig. 7E). Furthermore, the MDs in the treated embryos were enlarged relative to controls, by contrast to the effects of XFD (Fig. 3B). Crucially, there were no other indications of irregular somatic tissues. We cannot exclude the possibility that MAPK is required for the development of the somatic component of the genital ridge. However, these results, together with the induction of PGCs from animal caps, suggest that MAPK plays a specific role in the development of PGCs (Fig. 7F).

## DISCUSSION

The induction of PGCs from axolotl animal caps was first reported by Boterenbrood and Nieuwkoop over 40 years ago (Boterenbrood and Nieuwkoop, 1973), but the identity of the agents that stimulate the formation of PGCs has remained unknown. Here, we show that PGCs are induced by a combination of FGF and BMP signalling. FGF has been shown to act as an inducer of mesoderm in seminal studies that used *Xenopus* embryos (Kimelman and Kirschner, 1987; Slack et al., 1987); FGF is known to support posterior mesoderm development through the induction and maintenance of *brachyury* expression (Amaya et al., 1991; Fletcher and Harland, 2008). However, the pluripotent mesoderm that gives rise to PGCs is not conserved in frogs (Johnson et al., 2003b). Thus, it is not surprising that a role for FGF in germline development has gone unrecognised. We showed that FGF can redirect mesoderm that has been induced by Smad2 to form the germ line instead of blood. In addition, the forced expression of FGF inhibited the development of blood in the VMZ, as it does in *Xenopus* (Isaacs et al., 2007; Walmsley et al., 2008) (Fig. 3D). We suggest, therefore, that FGF promotes PGC development by inhibiting mesoderm differentiation. This is achieved through MAPK activity downstream of the *brachyury*-FGF feedback loop, and we postulate that MAPK signalling arrests the development of mesoderm precursors in the VMZ to maintain germ cell potential (Fig. 7F). How MAPK functions in this context is unclear; however, MAPK sustains *NANOG* expression in human embryonic stem cells (hESCs) (Yu et al., 2011), and axolotl animal caps (A.D.J., unpublished). Furthermore, recent work has demonstrated a central role for MAPK in the regulation of chromatin on pluripotency genes of both mouse and human ESCs (Goke et al., 2013; Tee et al., 2014). On this basis, we postulate a role for Elk-1, which can target MAPK to promoters (Zhang et al., 2008). Elk-1 has been shown to inhibit the differentiation of hESCs (Goke et al., 2013), and it might function to maintain germ cell potential downstream of *brachyury*. This, or related factors, might also later initiate germline commitment from undifferentiated precursors.

The original proposal that urodele PGCs are specified by induction, rather than by germ plasm, was controversial (Michael, 1984; Nieuwkoop and Sutasurya, 1979; Smith et al., 1983). Indeed, the possibility that two unrelated modes of PGC specification could exist in amphibians led Nieuwkoop and Sutasurya to propose that amphibians are diphyletic – i.e. from two distinct lineages of fish (Nieuwkoop and Sutasurya, 1976). However, it is now known that the urodele and anuran (frog) lineages of amphibians diverged from a common urodele-like ancestor over 260 million years ago (Ahlberg et al., 2005; Anderson et al., 2008; Zhang and Wake, 2009). Recent evidence indicates that, subsequent to this, *nanog* was deleted from the frog genome, and the gene regulatory network for mesoderm in *Xenopus* evolved to include an increased number of copies of the *nodal* and *mix* genes (Dixon et al., 2010; Hellsten et al., 2010; Swiers et al., 2010). Here, we show that Mix overexpression is sufficient to eliminate the PGCs in programmed animal caps and to stimulate the development blood. These results support the hypothesis that expansion of *mix* and *nodal* families was constrained in urodeles and other vertebrates that employ epigenesis, because increased Mix activity would terminate the germ line (Johnson et al., 2011; Swiers et al., 2010). Similarly, we also show that inhibition of MAPK signalling elicits a PGC-specific effect on axolotl development. Because MAPK inhibition results in gastrulation defects in *Xenopus*, but not axolotls, our results suggest that MAPK signalling was integrated into the gene regulatory network for frog mesoderm at a point after germ plasm had evolved and MAPK no longer participated in development of the germ line.

The end purpose of PGC specification is to produce cells that can become gametes. Surprisingly, it is clear that PGC development in the animal kingdom is initiated from a diverse array of upstream pathways. For example, in frogs, PGCs are of endodermal origin, whereas, in sea urchins, PGCs develop in a cell-autonomous manner from small micromeres (Yajima and Wessel, 2012). In this study, we demonstrate that axolotl PGCs develop downstream of the same pathway that initiates mesoderm development. Phylogenetic inference indicates that this is the primitive mechanism for PGC specification in terrestrial vertebrates, and probably vertebrates in general (Johnson et al., 2001, 2003b, 2011). Moreover, recent work on basal insects demonstrates that PGCs are derived from abdominal mesoderm by epigenesis, suggesting that a mesodermal origin for PGCs is basal to metazoans (Ewen-Campen et al., 2013). By contrast, direct programming of PGCs from mouse ESCs with transcription factors demonstrates that the mesodermal programme is dispensable for gamete production in the mouse (Magnusdottir et al., 2013; Nakaki et al., 2013). It is possible that *Blimp1* evolved as a target of *T* in rodents to accelerate germline restriction, and that this short circuited an ancestral mesoderm programme for initiating PGC development. A consequence of *Blimp1*, therefore, is that germline development in mouse is initiated from a quasi-pluripotent state (Leitch and Smith, 2013), rather than mesoderm. It is clear that the mechanism of PGC specification in urodeles was adapted as primitive amniotes and mammals evolved (Bachvarova et al., 2009a,b). We argue, therefore, that elucidation of this mechanism could aid in the development of *in vitro* methods for producing PGCs from non-rodent mammals, such as humans, because current methods for these species remain inefficient (Hayashi et al., 2012).

A major conclusion from this study is that irreversible commitment to the germ line in axolotls occurs after gastrulation is completed and the somatic cell lineages are established. Thus, the germ line is the last cell lineage to be established, contrasting starkly with other models. On this basis, we propose, the ‘last cell standing hypothesis’ in which PGCs are derived from the last cells in the embryo that are to undergo lineage commitment. This model postulates that, in the ancestral case, the germ line is a basal cell lineage, not one that is specialised by germline determinants. Predetermined germ cells evolved repeatedly in vertebrates, and their appearance is associated with embryological innovations that lead to accelerated development and an enhanced rate of evolution (Evans et al., 2014; Johnson et al., 2003a,b, 2011). Their prevalence in model systems has long obscured the complex evolutionary history of the germ line-soma relationship.

## MATERIALS AND METHODS

### Axolotl embryos and explants

Embryos were collected following natural matings as described previously (Johnson et al., 2001). For microinjection, embryos were manually de-jellied and cultured in 1× modified Barth's solution (MBS) with 4% Ficoll (Sigma). From stage 7, embryos were maintained in 0.2× MBS, and dissected explants were maintained in 1× MBS. Culture solutions were supplemented with antibiotics (50 µg/ml penicillin and streptomycin, and 20 µg/ml kanamycin) and fungizone (50 µg/ml). Embryos were staged as described previously (Bordzilovskaya et al., 1989). Activin A cell supernatant was diluted in 1× MBS supplemented with 0.1% bovine serum albumen fraction V (BSA V; Sigma). The Activin-*nodal* inhibitor SB431542 and FGF inhibitor SU5402 (Sigma) were solubilised in dimethyl sulfoxide and used at a final concentration of 50 μM.

### *In vitro* transcription and microinjection

mRNAs for microinjection were synthesised using mMessage mMachine (Ambion) from plasmids encoding *Xenopus* BMP4 (XBMP4) (Jones et al., 1992), *Xenopus* eFGF (Isaacs et al., 1994), *Xenopus* Smad2C (XSmad2C) (Howell and Hill, 1997), XFD (Amaya et al., 1991), axolotl Mix and axolotl Brachyury (Swiers et al., 2010).

### Lineage tracing

Mini-Ruby dextran (mini-Ruby; Invitrogen) was prepared, as described previously (Lane and Smith, 1999). Following *in situ* hybridisation on wax sections, mini-Ruby was detected by avidin-biotin-horseradish peroxidise conjugation using a Vectastain Elite ABC Kit (Vector Laboratories), followed by visualisation by using diaminobenzidine-hydrogen peroxide.

### Histology

For histological examination, embryos and explants were fixed in 2.5% glutaraldehyde in buffered sodium cacodylate, and 0.5 µm plastic sections were prepared and stained with Toluidine Blue or H&E using standard methods.

### *In situ* hybridisation

Embryos were fixed in phosphate-buffered 4% paraformaldehyde for at least 48 h. ISH was performed on sections of embryos and larvae using digoxigenin-labeled probes for axolotl *dazl* (Johnson et al., 2001) and axolotl *globin* (AF308869), as described previously (Johnson et al., 2001).

### Quantitative PCR

RNA was extracted and analysed by qRT-PCR (Taqman or SYBR Green probes), as described previously (Swiers et al., 2010). The oligonucleotide sequences for qRT-PCR are listed in supplementary material Table S1. All error bars indicate 95% confidence intervals (CIs).

### Deep sequencing

Libraries prepared from uninjected caps were compared with caps that had been injected with FGF and BMP by Illumina RNA-sequencing (carried out at The Genome Centre, Queen Mary, University of London, London, UK). Approximately 50 million paired-end reads were generated for each sample. These reads were mapped to an in-house reference collection of axolotl transcripts using Bowtie (Langmead et al., 2009). The reference collection was annotated using Blast2Go and transcripts that were associated with PGC specification were identified by GO annotation (Conesa et al., 2005). Differential expression of transcripts was determined using DEGseq (Wang et al., 2010) with a fourfold minimal change between samples.

## Supplementary Material

Supplementary Material
