# Supplementary material for: Stochastic specification of primordial germ cells from mesoderm precursors in axolotl embryos
Source: Development. 2014 Jun;141(12):2429–40. doi: 10.1242/dev.105346 (PMC4050694; doi:10.1242/dev.105346)
Supplement: Supplementary Material [file supp_141_12_2429__index.html]

Stochastic specification of primordial germ cells from mesoderm precursors in axolotl embryos — Supplementary Material 

# Stochastic specification of primordial germ cells from mesoderm precursors in axolotl embryos

## DEV105346 Supplementary Material

**Files in this Data Supplement:**

- **Supplementary Material**
