# Supplementary material for: Stochastic specification of primordial germ cells from mesoderm precursors in axolotl embryos
Source: Development. 2014 Jun;141(12):2429–40. doi: 10.1242/dev.105346 (PMC4050694; doi:10.1242/dev.105346)
Supplement: Supplementary Material [file supp_141.12.2429_DEV105346.pdf]

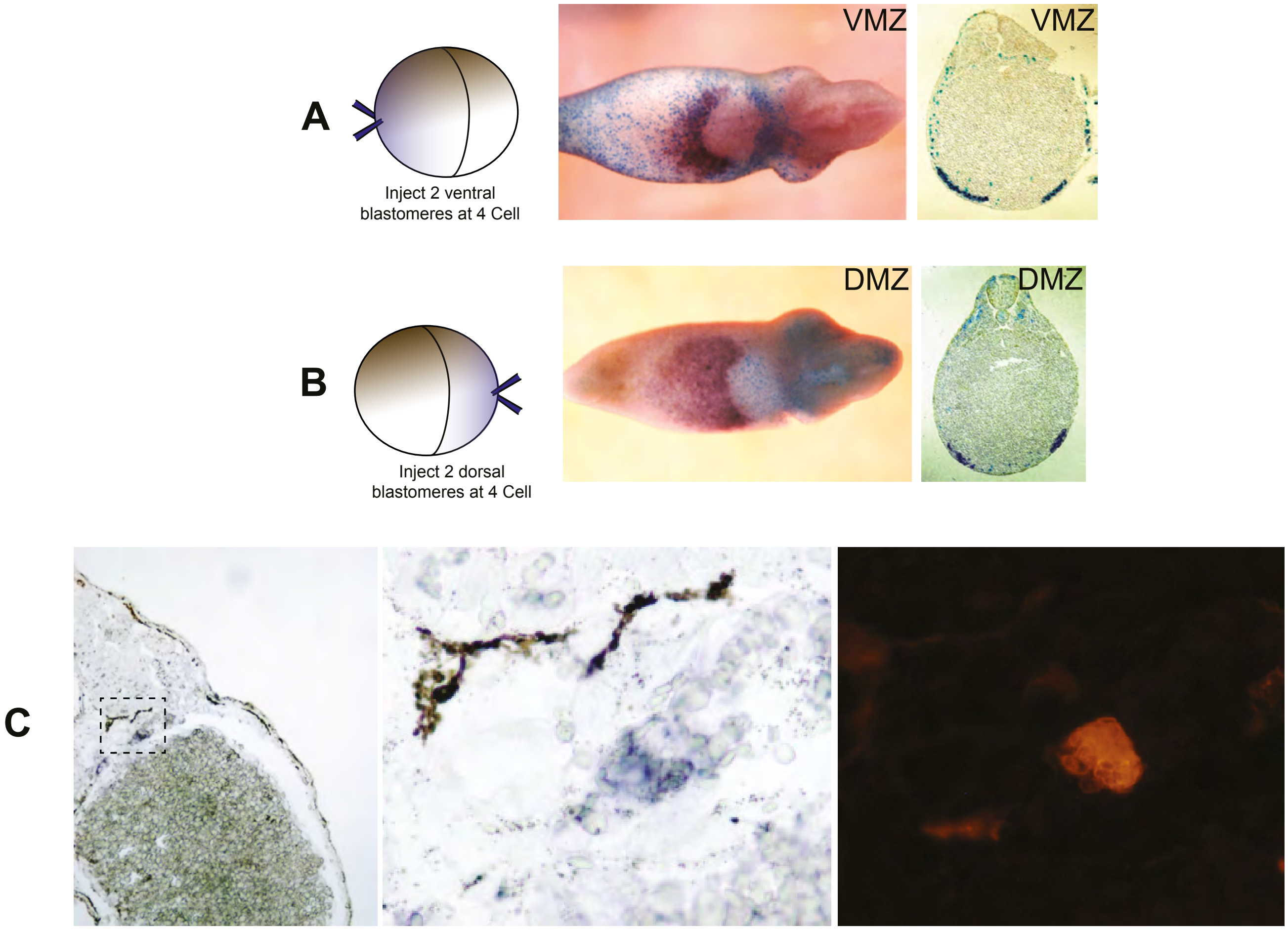

Figure S1. Lineage tracing in the axolotl VMZ. (A) Embryos were injected at 4 the cell stage into the ventral marginal zone (VMZ) with RNA for lacZ (left) as described in Ciau-Uitz et al. (2000). Tailbud stage embryo were stained with X-gal and then used in whole mount in situ hybridization to detect *globin* RNA. Ventral view (middle) shows overlap of Xgal staining (blue) and *globin* RNA (purple) and section (right) shows overlapping signal. (B) Same as in A, except that dorsal marginal zone was injected. (C) ISH to detect *dazl* RNA on section from stage 42 embryo (Left) in which one lateral blastomere was injected with miniruby at 128 cell stage. Enlarged bright view (Middle) of boxed region containing PGC cluster. PGC cluster under UV light shows miniruby (Right).

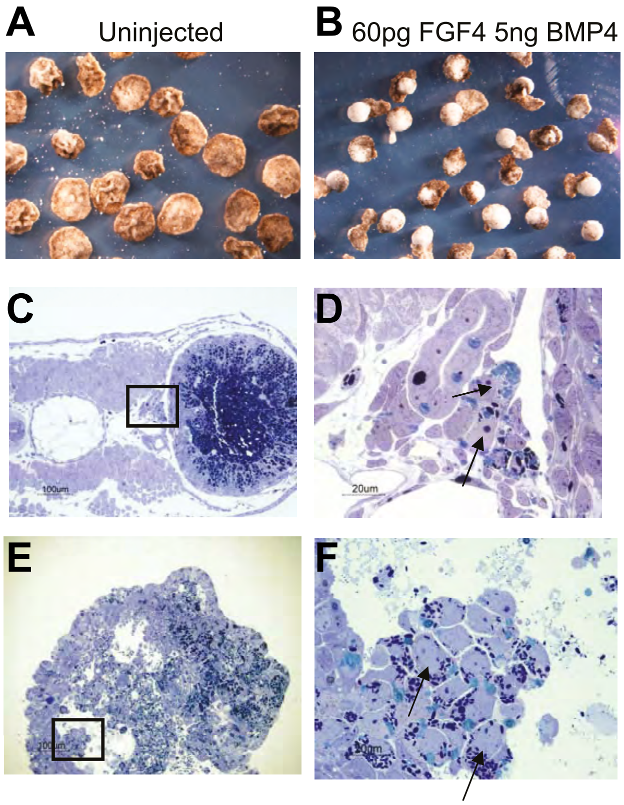

Figure S2. Histological analysis of PGCs induced by FGF and BMP-4. (A) Animal caps from uninjected embryos at stage 42 of development. (B) Animal caps from embryos injected with RNA for FGF (60pg) and BMP-4 (5ng). Note large white mass, which contains the PGCs. (C) Section of stage 45 embryo stained with toluidine blue. AGM region is highlighted in hatched box. (D) High magnification view of the AGM region from embryo in C. Arrows point to PGCs. Note large nucleus and single prominent nucleolus. (E) Section from animal cap induced with FGF and BMP-4. Boxed region shows cluster of PGCs. (F) Closeup view of PGCs (arrows) identified in E. Note the large nucleus and single prominent nucleolus.

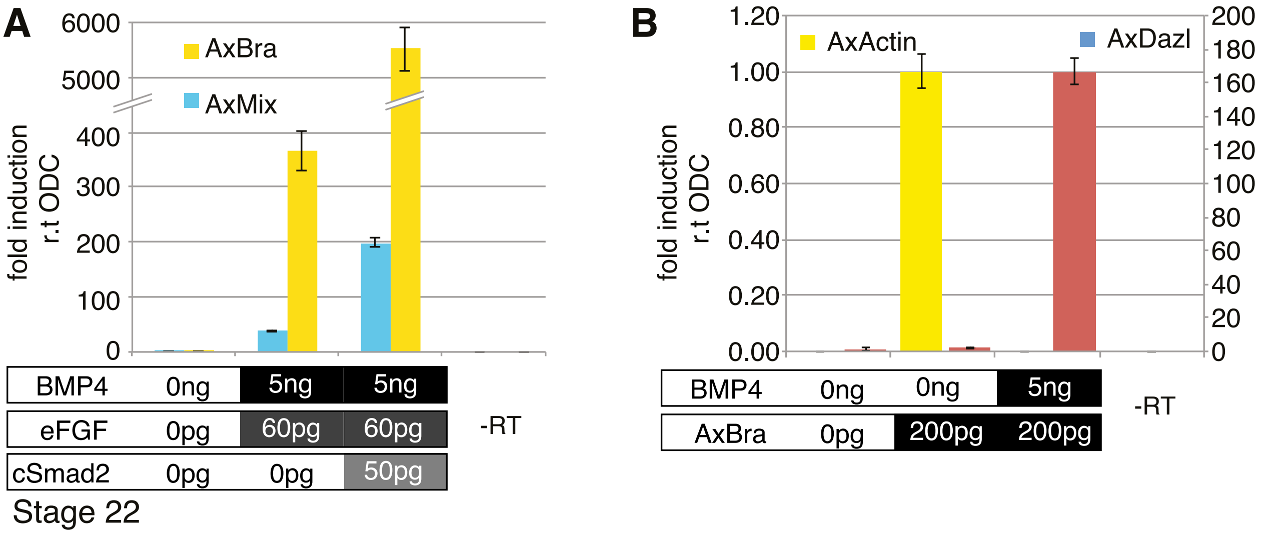

Figure S3. Brachyury induction in axolotl animal caps. (A) qPCR analysis of *Brachyury* and *Mix* induction in animal caps after ventral mesoderm induction. Smad2C increases *brachyury* expression almost 15 fold over FGF alone. (B) qPCR analysis of α-*actin* and *dazl* expression after induction of PGCs by brachyury and BMP-4. Brachyury and BMP-4 induce *dazl* but not α-*actin* expression in animal caps. Brachyury on its own induces α-*actin.*

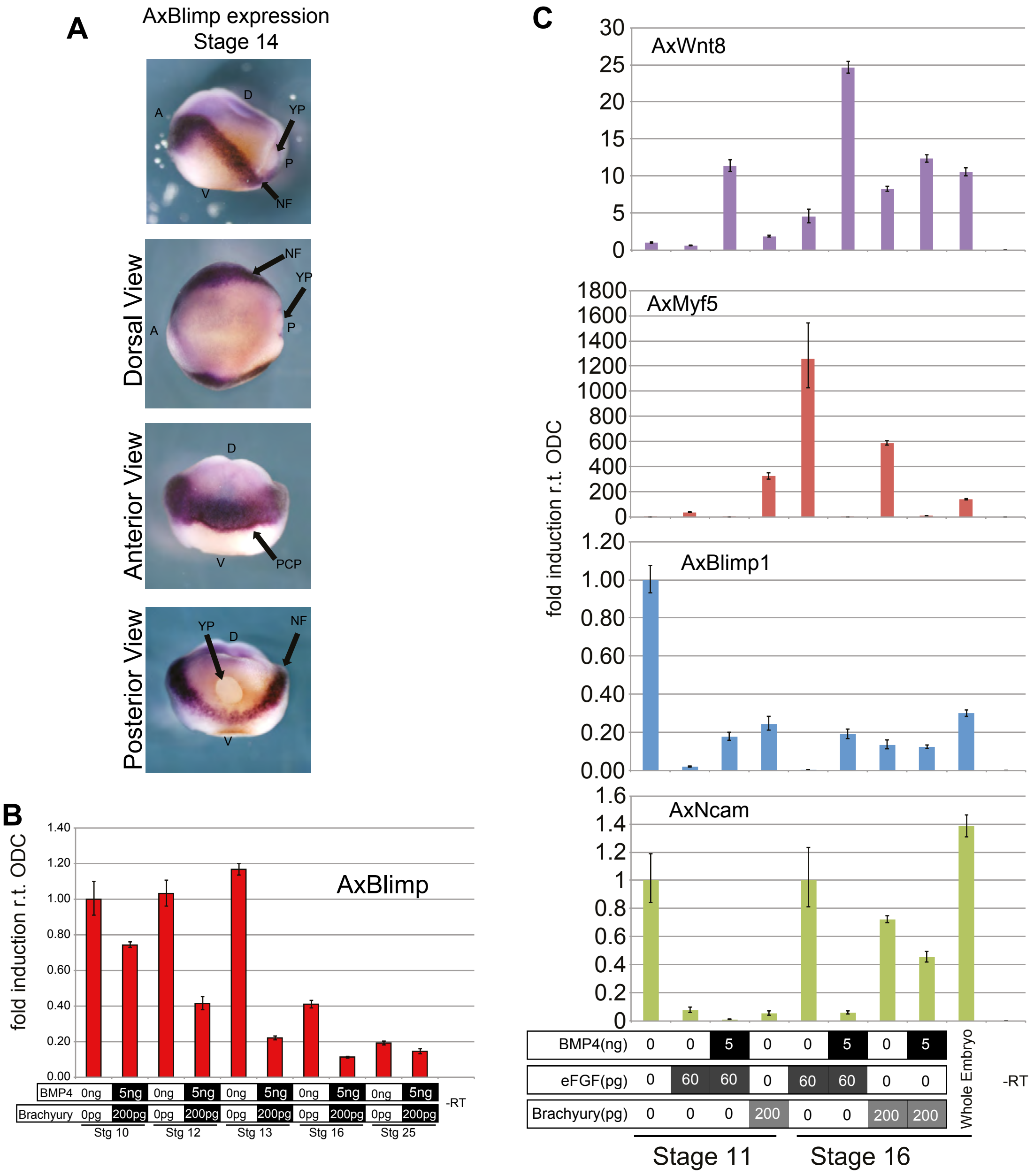

Figure S4. *blimp1* expression in early axolotl embryos. (A) Whole mount in situ analysis of Blimp1 expression in early neurulae. Zygotic expression is detected in neural folds (NF). Expression is not detected in the posterior lateral mesoderm domain that will give rise to PGCs. (A, anterior, P, posterior, D, dorsal, V, ventral, YP= yolk plug, PCP= prechordal plate). (B) time course analysis comparing *blimp1* expression in uninjected animal caps with caps programmed with RNA for Brachyury and BMP-4. Brachyury and BMP-4 do not induce *blimp1* expression. (C) qRT-PCR analysis of *Blimp1* and neural (*ncam*) or mesoderm (*myf*-5, *wnt*-8) markers in animal caps in response to induction by FGF or Brachyury, with or without BMP-4 RNA.

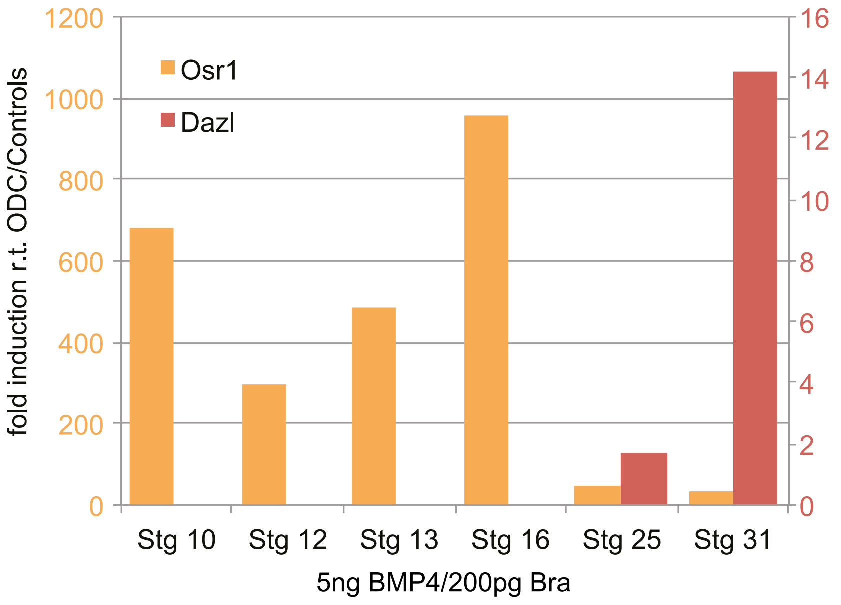

Figure S5. Down-regulation of *osr-1* coincides with germ line restriction. RNA from a time course of animal caps programmed with RNA for Brachyury and BMP-4 was analysed for expression of *osr1* and *dazl* by qRT-PCR. *osr1* expression begins to extinguish at about tailbud stage (stage 25), when low levels of *dazl* expression first become detectable.

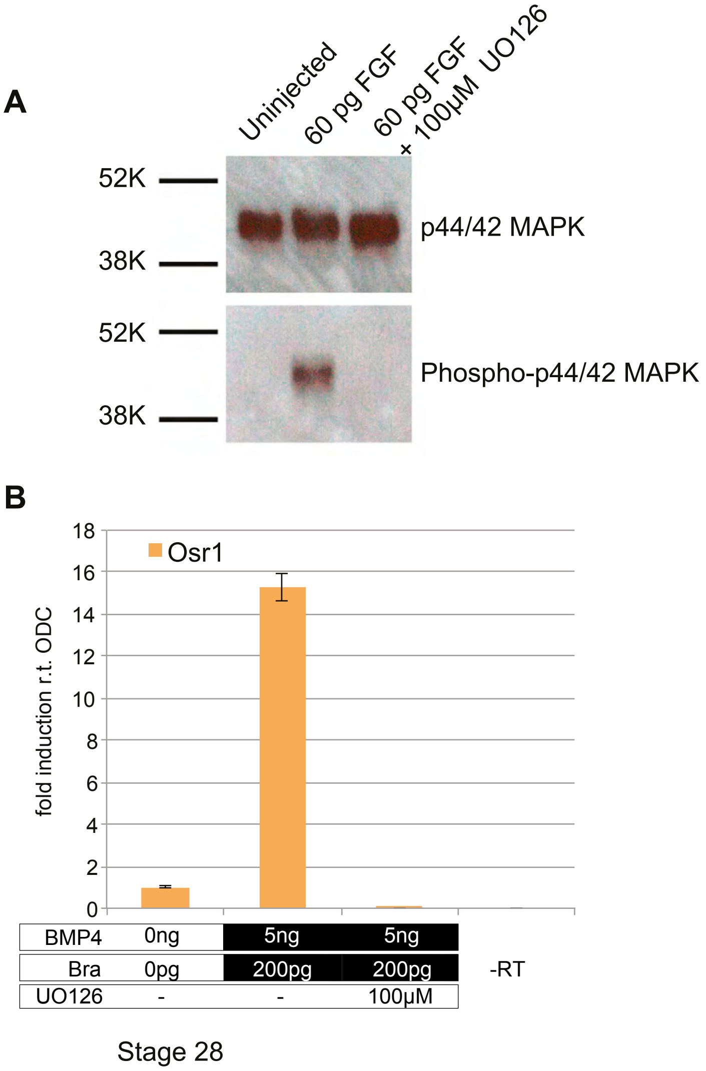

Figure S6. MAPK signalling maintains *osr1* expression in intermediate mesoderm. (A) Western blot to detect the effects of UO126 on programmed animal caps. Animal caps were dissected from uninjected embryos, or embryos injected with FGF RNA (60pg). Some of the caps from injected embryos were cultured in UO126 (100µM). All caps were incubated overnight at 20°C before harvesting. Western blots were probed to detect the unphosphorylated or phosphorylated (Phospho-p44/42 MAPK) forms of MAPK. (B) qRT-PCR analysis of RNA from animal caps to detect *osr1*. Animal caps were either uninjected or were programmed with RNA for Brachyury and BMP-4. Some injected caps were incubated from the midblastula through gastrula stages in UO126 (100µM). Caps were harvested at stage 28 (midtailbud) stage.

Supplementary Table 1

A list of PGC molecules overexpressed in animal caps at stage 42 injected with FGF and BMP-4 mRNA. Key PGC markers are highlighted in bold. Note that the bone morphogenetic protein 4 detected (italics) represents the exogenous BMP-4.

**Up-Regulated In PGCs**	**GO:Annotation**
3-hydroxy-3-methylglutaryl- reductase	GO:0008354
4snc-tudor domain protein	GO:0007281
activin receptor type-1 precursor	GO:0007281 GO:0008354
**adenosine deaminase domain containing 1 (testis specific)**	GO:0002314 GO:0002636
adp-ribosylation factor-like 5c	GO:0051729
ankyrin sam and basic leucine zipper domain containing 1	GO:0007281
apoptosis regulator bcl-x	GO:0007281
atp-dependent rna	GO:0007281 GO:0040022
bone morphogenetic protein 4 (likely exogenous mRNA)	GO:0007281
brunol5 protein	GO:0007281
cadherin neuronal	GO:0008354
cadherin type m-cadherin	GO:0008354
ccr4-not transcription complex subunit 8	GO:0051729
cdh1 protein	GO:0008354
chemokine receptor 4	GO:0007281 GO:0008354
**daz interacting protein 1-like**	Manually Identified
**daz-like protein**	GO:0007281 GO:0008354
dead (asp-glu-ala-asp) box polypeptide 59	GO:0007281 GO:0040021
dead box atp-dependent rna	GO:0051729
**dead end homolog 1**	GO:0007281
degenerative spermatocyte homolog lipid desaturase	Manually Identified
delta 1	GO:0007281
dhx33 protein	GO:0007281 GO:0040022
dicer-1 cg4792-pa-like protein	GO:0042078
dna repair protein rad51	GO:0043073
dna-dependent protein kinase catalytic subunit	GO:0035234
**vasa**	Manually Identified
e-cadherin	GO:0008354
epsin 3	GO:0051729
eukaryotic initiation factor 4a	GO:0051729
f-box and wd-40 domain protein 7	GO:0040022
fanconi anemia complementation group c	GO:0007281
fem1c protein	GO:0019100 GO:0042006
frl- cryptic family 1	GO:0001704 GO:0008354
g-protein coupled receptor	GO:0008354
**germ cell nuclear factor**	Manually Identified
**germ cell-less homolog 1**	Manually Identified
glutamate receptor interacting protein 2	GO:0007281 GO:0008354
glypican 1	Manually Identified

gtp-binding adp-ribosylation factor homolog 1 protein	GO:0051729
guanine nucleotide binding 3	GO:0051729
heterochromatin protein binding protein 3	GO:0001674
histone deacetylase 1	GO:0043073
histone h2a	GO:0001673
homolog 2 (coli)	GO:0007281
integrin beta-1 precursor	GO:0008354
karyopherin alpha 3	GO:0040022
keratin 7	Manually Identified
kh-domain rna binding	GO:0040022 GO:0042006 GO:0051729
lactate dehydrogenase b	Manually Identified
lactate dehydrogenase c	Manually Identified
lactate dehydrogenase d	Manually Identified
**lin-28 homolog b**	Manually Identified
lipid phosphate phosphohydrolase 1	GO:0008354
**maelstrom homolog**	Manually Identified
**mago-nashi-like protein**	GO:0040022
marcks-like 1	GO:0042585
microtubule-associated protein 7 domain containing 1	GO:0007281
mismatch repair protein	GO:0001673
mkiaa4096 protein	GO:0007281 GO:0040022
moloney leukemia virus 10-like homolog	GO:0007281
**motile sperm domain containing 2**	Manually Identified
**mpdz protein**	GO:0007281 GO:0008354
myristoylated alanine-rich c-kinase substrate	GO:0042585
novel krab box and zinc c2h2 type domain containing protein	GO:0007281
novel protein*	GO:0042078 GO:0043073
novel protein vertebrate moloney leukemia virus homolog	GO:0007281
novel protein zebrafish epithelial cadherin 1	GO:0008354
**nuclear autoantigenic sperm protein (histone-binding)**	Manually Identified
nucleolar protein of 40 kda	GO:0007281 GO:0040022
peptidylprolyl isomerase -like 2	GO:0040022
phospholipase d2	GO:0010004
**piwi-like 1**	GO:0030718
**piwi-like 2**	GO:0007281 GO:0030718
placental protein 11 related	Manually Identified
platelet derived growth factor receptor alpha	GO:0007281 GO:0035234
pol polyprotein	GO:0007281
**poly polymerase beta (testis specific)**	Manually Identified
**pr-domain containing protein 9**	GO:0007281
**prdm1 protein**	Manually Identified
proteasome alpha subunit	GO:0040022
rdc1 like protein	GO:0008354
replication protein a1	GO:0001673
rna binding homolog 1	GO:0007281 GO:0008354

rna binding protein bruno-like 3b	GO:0007281
rna helicase	GO:0007281 GO:0007294 GO:0030720 GO:0040021
rna-binding protein 8a	GO:0040022
**round spermatid basic protein 1-like**	Manually Identified
sdad1 protein	GO:0051729
serine threonine kinase	GO:0007281
serologically defined colon cancer antigen 10	GO:0040022
smad1 5	GO:0007281 GO:0030718
**sperm associated antigen 5**	Manually Identified
**sperm associated antigen 6**	Manually Identified
**sperm tail associated protein**	Manually Identified
**sperm-associated antigen 7**	Manually Identified
**spermatogenesis-associated protein 2**	Manually Identified
**spermidine spermine n1-acetyltransferase family member 2**	Manually Identified
**spermidine synthase**	Manually Identified
**spermine oxidase**	Manually Identified
splicing factor u2af 65 kda subunit	GO:0007281
**stromal antigen 2**	GO:0001673
**stromal antigen 3**	GO:0001673
**stromal cell-derived factor 1 precursor**	GO:0007281
superkiller viralicidic activity 2-like 2	GO:0008354
target of rapamycin	GO:0007281
**tdrd5 protein**	GO:0007281
**telomerase-associated protein 1**	Manually Identified
telomeric repeat binding factor 1	GO:0001673
**testis derived transcript (3 lim domains)**	Manually Identified
**testis expressed 10**	Manually Identified
**testis expressed 2**	Manually Identified
thyroid hormone receptor interactor 13	GO:0001673
transforming growth factor-beta	GO:0008354
trinucleotide repeat containing 4	GO:0007281
**tudor domain containing 6 protein**	GO:0007281
**tudor domain containing 9**	GO:0007293 GO:0007294 GO:0030720
**tudor repeat 1 protein**	GO:0007281
type i serine threonine kinase receptor	GO:0008354
zinc finger protein 182	GO:0007281
**zona pellucida 2 glycoprotein**	Manually Identified

*Multiple proteins were annotated as “novel protein” by blast2go. Of these a subset were annotated with GO terms suggesting a role in PGC specification and maintenance.

Supplementary Table 2

Real-time PCR Primers (Taq Man)

Gene	Forward Primer (5'-3')	Reverse Primer (5'-3')	Probe (5'-3')
**axblimp**	CGATGGTGACTCCGAAACAGA	GGCACTTTTGTGGTGAAGCA	AACCCAAAGCAACAGCACACTGAAAAGG
**axbra**	CATTGACCACATGTACCAATTGC	GATCAAGGGTCAATCGTGAGTTC	TACCCATAGTTCTTTTGTGCAGCATCCACG
**axdazl**	CATGAACCTGCTCAGCCATCT	GCAGGATACGACTGTCTGAATGC	CTGGAAACAGCCCTCAAAAAAAATCTGTGG
**axflk1**	GACTCAGAAAAGACACTG	GCAACTTGATCTTGTAATAAC	ACACTTGTCTGTTCTGGCTCAAC
**axglobin**	CATGGCGGTAAGGTTCTGAAC	CCCCATGTCGAGATCATCAAT	CCGTAGGTGAAGCCGCCAAGCA
**axmix**	GTCCAGGATCCAGGTCTGGTT	GCTTCTGGGTGGATTTGATTTATAA	AATAGGCGTGCCAAGTCCCGCC
**axncam**	TGAATGTCGTTCAACGTGAGAGA	AAGAAAAGACTCTGGATGGACGTATC	AGACCCTGGCGTGACTGCTCACCA
**axodc**	ATGCCCGTCATGAGTAGTACCA	CCCGGACCCAGGTTACG	TGACAGTTCCAAGGTTTCATTCAATTGCTG
**axosr**	CACCAAGTCTTACAACCTGTTG	CTTGGAGTGGATGTATCTGTG	CCACAAAGCATTCAGAAGGCAGG
**axpax2**	GAAGGCATCTGTGACAAC	GCTGGACTTTGGTTCTG	TCACAGATGCCTTCCGCCAG
**axpiwi**	AGCTTCGTTAAGAGCTTGGTTC	CGCCATCACGGTAGACAATG	TTTAACAATGCAATGCCTGCCCGG
**axvasa**	GATCGAATGCTTGATATGGGTTT	TGTTTGCCGTTCTTCTTTGGT	AAGACGTTAGTCACCAGTCCAGGAATGCC

Real-time PCR Primers (SYBR Green)

Gene	Forward Primer (5'-3')	Reverse Primer (5'-3')
**axwt1**	TCTCGATCTGATCATTTGA	GCAGTTTGGTCATATTCC
**axfli1**	CTCGTCATTGGGTTACAG	GAGGGATCTTCTTTGGAA
